# TRENDS OF ORAL HEALTH STATUS OF OLDER ADULTS BY IMMIGRATION STATUS IN THE UNITED STATES: 1999–2018

**DOI:** 10.1093/geroni/igae098.1427

**Published:** 2024-12-31

**Authors:** Huabin Luo, Bei Wu, Xiang Qi, Mark Moss

**Affiliations:** East Carolina University, Greenville, North Carolina, United States; New York University, New York, New York, United States; New York University, New York, New York, United States; East Carolina University, Greenville, North Carolina, United States

## Abstract

National data on oral health of immigrants in the United States is limited. This study assessed the oral health trends of this population from 1999 to 2018, using data from the National Health and Nutrition Survey. The outcome variable—self-reported oral health—was categorized as a binary outcome: excellent/very good/good or fair/poor. The sample was 18,853 adults aged 50 years and older, including 14,154 US-born Americans, 2,980 naturalized citizens, and 1,719 noncitizens. Trends in self-reported fair/poor oral health across these groups were identified. The findings revealed a significant decline in the prevalence of fair/poor oral health among all groups between 1999 and 2018 (P for trend <.05). However, noncitizens consistently reported higher rates of fair/poor oral health compared to US-born and naturalized citizens. In the logistic regression model only controlling demographics, noncitizens were more likely (AOR=1.28) and naturalized citizens were less likely (AOR=0.80) to report fair/poor oral health. After further adjustment for education, income, and smoking status, the disparities in fair/poor oral health among the groups were not statistically significant. This study found an overall decrease in self-reported fair/poor oral health among older adults in the US during 1999-2018. However, significant oral health disparities persisted, particularly for noncitizens. Further research is needed to validate these trends and develop targeted interventions to address the oral health disparities among older immigrants in the US.

